# Causes and Contributing Factors of In‐Hospital and Post‐Discharge Mortality Among Patients With Severe Acute Respiratory Infection in Low‐ and Middle‐Income Countries: A Scoping Review

**DOI:** 10.1002/hsr2.72927

**Published:** 2026-09-13

**Authors:** Md Ariful Islam, Holly Seale, Md Saiful Islam, Saju Bhuiya, Md Zakiul Hassan, Quazi Fayeza Sultana, Fahmida Chowdhury, Abrar Ahmad Chughtai

**Affiliations:** ^1^ School of Population Health, Faculty of Medicine and Health University of New South Wales Sydney New South Wales Australia; ^2^ Infectious Diseases Division, International Centre for Diarrhoeal Disease Research, Bangladesh (icddr,b) Dhaka Bangladesh

**Keywords:** causes, factors, LMIC, mortality, scoping review, severe acute respiratory infection

## Abstract

**Background and Aims:**

Severe acute respiratory infections (SARI) remain a major cause of global morbidity and mortality, with a disproportionate burden in low‐ and middle‐income countries (LMICs). Although in‐hospital mortality is frequently reported, deaths occurring after hospital discharge remain poorly documented. We aimed to synthesise the available evidence on the causes and contributing factors of in‐hospital and post‐discharge mortality among patients hospitalised with SARI in LMICs.

**Methods:**

We conducted a scoping review following the Arksey and O'Malley framework and PRISMA‐ScR guidelines. PubMed, Web of Science, Scopus, and CINAHL were searched for studies published between January 2000 and October 2025 that reported in‐hospital and/or post‐discharge mortality among patients with SARI in LMICs. Findings were synthesised narratively to describe factors associated with in‐hospital and post‐discharge mortality, as well as reported causes of death.

**Results:**

Twenty‐nine studies from 13 LMICs met the inclusion criteria. All reported in‐hospital mortality (range: 0.4%–39.3%), while four reported post‐discharge mortality (range: 0.6%–5.7%). Thirteen studies examined factors associated with mortality. In‐hospital mortality was associated with older age, male sex, comorbidities, markers of severe clinical presentation, specific respiratory pathogens, and delayed healthcare seeking. Only one study specifically evaluated factors associated with post‐discharge mortality, identifying respiratory difficulty, underlying heart disease, and longer hospital stay among children, and chronic obstructive pulmonary disease and intensive care unit admission among adults as risk factors. Pathogen‐specific causes of in‐hospital death were reported in a single study, and no study reported definitive causes of post‐discharge mortality.

**Conclusion:**

Evidence on SARI‐associated mortality in LMICs is largely confined to in‐hospital outcomes, with limited assessment of post‐discharge mortality or causes of death. Standardised post‐discharge follow‐up and systematic cause‐of‐death reporting are needed to inform clinical care, strengthen surveillance, and support targeted interventions to reduce preventable SARI‐associated mortality in LMICs.

**Abbreviations**
aHR: adjusted hazard ratio
aOR: adjusted odds ratio
ARDS: acute respiratory distress syndrome
CI: confidence interval
hMPV: human metapneumovirus
HR: hazard ratio
ICU: intensive care unit
IQR: interquartile range
OR: odds ratio
qSOFA: quick Sequential Organ Failure Assessment
RSV: respiratory syncytial virus
SARI: severe acute respiratory infection
SD: standard deviation
SpO_2_
: peripheral oxygen saturation

## 1 Introduction

Severe acute respiratory infections (SARI) pose a major global public health challenge and are a leading cause of hospitalisation, intensive care admission, and death worldwide, particularly among young children, older adults, and people with underlying medical conditions [1, 2, 3]. SARI accounts for an estimated 4.2 million deaths annually, with up to 90% of deaths occurring in low‐ and middle‐income countries (LMICs) [2, 4, 5, 6]. The high mortality among patients with SARI in resource‐limited settings reflects a complex interplay of demographic factors, constrained access to healthcare, and socioeconomic inequalities [5]. The COVID‐19 pandemic further intensified the impact of SARI, underscoring the need to better understand the factors contributing to fatal outcomes in LMICs [7].

SARI can result from a diverse range of infectious agents, including respiratory viruses, bacteria, and other microorganisms, with respiratory viruses representing the leading causes [8]. Common viral aetiologies include influenza viruses, SARS‐CoV‐2, respiratory syncytial virus (RSV), and other emerging respiratory pathogens [8, 9]. Several viral pathogens, particularly influenza A(H1N1)pdm09 and SARS‐CoV‐2, have demonstrated the capacity to cause large, rapidly spreading outbreaks with substantial health and socioeconomic consequences worldwide [10, 11]. SARI and SARI‐related deaths may also result from co‐infection with multiple pathogens [8, 12]. Recent studies have shown that co‐infections involving two or more respiratory pathogens were common during the COVID‐19 pandemic [13]. Common viral co‐infections included influenza, rhinovirus or enterovirus, human metapneumovirus, and RSV [14]. The most frequently reported bacterial co‐infections during COVID‐19 included Mycoplasma pneumoniae, Chlamydophila pneumoniae, Staphylococcus aureus, and Streptococcus pneumoniae [15].

Mortality among hospitalised patients with SARI occurs either during admission or after discharge, representing distinct but interrelated risk phases [16]. In‐hospital mortality refers to deaths during the index hospitalisation, reflecting acute disease severity, clinical complications, and inpatient care quality [17]. In contrast, post‐discharge mortality refers to deaths among patients discharged alive who subsequently die, often within weeks, due to unresolved illness, post‐acute complications, underlying comorbidities, or gaps in continuity of care [16].

While in‐hospital mortality among patients with SARI is frequently reported, deaths occurring after hospital discharge remain largely undocumented in LMICs [16]. Emerging evidence indicates that post‐discharge mortality may contribute substantially to overall mortality among patients with SARI [16, 18]. The existing literature is fragmented, with most studies focusing on single pathogens, individual healthcare facilities, or specific age groups, thereby limiting a comprehensive assessment of mortality determinants [1, 19, 20]. To date, no comprehensive review has yet synthesised evidence across diverse healthcare settings, pathogens, and populations to examine both in‐hospital and post‐discharge SARI mortality in LMICs.

This scoping review synthesises available evidence to identify factors and causes associated with both in‐hospital and post‐discharge mortality among patients with SARI in LMICs, with the aim of informing clinical practice, identifying research priorities, and guiding the development of effective interventions.

## 2 Methods

### 2.1 Scoping Review Framework

We conducted this scoping review following the Arksey and O'Malley methodological framework [21] and adhered to the Preferred Reporting Items for Systematic Reviews and Meta‐Analyses extension for Scoping Reviews (PRISMA‐ScR) checklist [22] to ensure methodological rigour and transparency. Two research questions guided the review: (1) what factors are associated with in‐hospital and post‐discharge mortality among patients with SARI in LMICs, and (2) what causes of in‐hospital and post‐discharge deaths have been reported among this population. This scoping review was registered with the Open Science Framework (https://osf.io/uvmpx).

## 3 Eligibility Criteria, Data Sources, and Literature Search Strategy

We defined the eligibility criteria a priori (Table 1, Supporting Information Table S1). We conducted a comprehensive search of Web of Science, PubMed, Scopus, and CINAHL for articles published between January 2000 and October 2025. We developed the PubMed search strategy using Medical Subject Headings (MeSH) and adapted it for the other databases. We imported all records into Covidence for deduplication and screening [23]. We completed the electronic database searches on 1 November 2025. Two reviewers (M.A.I. and S.B.) independently screened titles, abstracts, and full texts and extracted data; a third reviewer (QFS) resolved any disagreements. We synthesised the data using descriptive and narrative approaches to compare findings across studies, under the supervision of AAC, HC, and MSI.

**TABLE 1:** Search strategy for articles on mortality among hospitalised patients with severe acute respiratory infection in low‐ and middle‐income countries.

Search strategy item	Description
Databases	PubMed, Scopus, Web of Science, and CINAHL
Timeframe	January 2000–October 2025
Language	English language or with English translation
Geographical scope	Low‐ and middle‐income countries (LMICs)
Core keywords	Severe acute respiratory infection AND mortality AND low‐ or middle‐income country
Search strategy	(‘severe acute respiratory infection’) AND (‘death’ OR ‘mortality’ OR ‘fatal outcome’) AND (‘low‐ and middle‐income countries’ OR ‘LMICs’ OR ‘resource‐limited settings’)
Inclusion criteria	Original peer‐reviewed observational studies (cross‐sectional, case–control, or cohort studies), or randomised controlled trials reporting in‐hospital and/or post‐discharge mortality among patients with SARI in LMICs
Exclusion criteria	Reviews, abstracts, commentaries, editorials, letters, expert opinions, or preprints; studies without original SARI mortality data; and studies conducted outside LMICs or including non‐LMIC populations in multicountry analyses or beyond the review scope

## 4 Inclusion Criteria

We included studies if they were original observational studies (cross‐sectional, case–control, or cohort), or randomised controlled trials conducted in LMICs, as defined by the World Bank 2026 criteria [24]. Eligible studies were published between January 2000 and October 2025 to capture the contemporary era of SARI surveillance, including major respiratory epidemics and pandemics such as influenza A(H1N1)pdm2009 and COVID‐19. Studies had to be available in English or with an English translation; include hospitalised patients meeting the WHO‐SARI case definition or a recognised national or regional adaptation; and report in‐hospital and/or post‐discharge mortality, associated factors, or causes of death among patients with SARI (Table 1).

## 5 Exclusion Criteria

We excluded conference abstracts, reviews, commentaries, editorials, letters, expert opinions, and preprints. We also excluded studies that did not report original mortality data among patients with SARI (e.g., studies reporting only modelled or estimated SARI mortality), studies conducted outside LMICs, and multicountry analyses that included non‐LMIC populations or otherwise fell outside the scope of this review (Table 1).

## 6 Definition of SARI Cases

The WHO SARI case definition, initially introduced in 2005 and revised in 2011, was further refined in 2014 [25, 26, 27, 28, 29]. As of January 2014, the WHO defines a SARI case as an acute respiratory infection with a history of fever or measured temperature ≥ 38°C and cough, with symptom onset within the last 10 days, and requiring hospitalisation [28]. This definition remains the WHO standard despite the emergence of COVID‐19 and relies on clinical criteria rather than laboratory confirmation of a specific pathogen. The 2014 SARI criteria have been widely adopted globally [30, 31, 32]. However, some countries and surveillance platforms have implemented slightly narrower or broader definitions [1, 33, 34, 35, 36] (Supporting Information Table S2). This review included studies that applied either the WHO SARI definition or country‐specific or locally adapted definitions.

## 7 Mortality Outcome Classification and Post‐Discharge Follow‐up Period

This scoping review included original studies that reported mortality outcomes among patients with SARI. Mortality outcomes were charted and categorised as in‐hospital mortality, defined as deaths occurring during the index hospital admission, and post‐discharge mortality, defined as deaths occurring within 4 weeks (1 month) after hospital discharge. Post‐discharge mortality was ascertained 4–6 weeks after discharge, as reported in the primary studies. Only patients who were discharged alive following their initial hospital admission were considered at risk for post‐discharge mortality, and outcome definitions were retained as reported in the included studies.

## 8 Data Extraction

We charted data from each eligible study using a structured, pre‐piloted data extraction form developed a priori in accordance with the review objectives. Two reviewers (M.A.I. and S.B.) independently extracted data, with disagreements resolved by a third reviewer (Q.F.S.). Core study data and contextual and methodological characteristics, including the SARI case definition used (WHO or other specified definition), country, study design and period, sample size, and data sources, were extracted. This structured data charting process facilitated narrative synthesis and comparison across LMIC settings.

## 9 Factors Associated With Mortality

We extracted patient‐level, health‐seeking, and health‐system factors analysed in relation to SARI mortality. Patient‐level variables included demographic characteristics (age and sex); underlying conditions (comorbidities and smoking history); clinical presentation and severity markers (severe clinical signs, advanced ventilatory support, critical illness complications, inflammatory blood biomarkers, and severity scores); radiological abnormalities (chest X‐ray findings); and respiratory pathogen aetiology based on laboratory confirmation. Health‐seeking factors included delay from symptom onset to hospital admission. Health‐system factors included healthcare settings and care‐related indicators. Studies that conducted regression‐based analyses (univariable or multivariable) were considered to have identified factors associated with SARI mortality. Studies reporting only descriptive characteristics of deceased patients without comparative statistical analyses were excluded, as such analyses cannot establish associations. When both univariable and multivariable analyses were reported, factors identified in multivariable models were extracted. Independent predictors identified through multivariable analyses were synthesised as factors associated with mortality, consistent with the objectives of this scoping review.

## 10 Cause of Death Recording

We extracted cause‐of‐death information as reported, including ascertainment methods (physician‐assigned diagnoses, hospital records, or laboratory‐confirmed pathogen identification). We documented whether studies used formal medical certification of cause of death (MCCD) or alternative approaches such as verbal autopsy, recognising that some studies reported pathogen attribution rather than underlying causes of death [37, 38, 39]. Owing to heterogeneity in ascertainment and reporting across LMIC settings, cause‐of‐death data were extracted as reported without reclassification and interpreted in light of differences in diagnostic and documentation practices.

## 11 Data Analysis

Extracted data were synthesised using descriptive and narrative approaches. We first summarised reported in‐hospital and post‐discharge mortality among patients with SARI in LMICs. We then synthesised evidence on factors associated with in‐hospital and post‐discharge mortality, analysed separately. Finally, we mapped the causes of death reported for in‐hospital and/or post‐discharge deaths among patients with SARI. Continuous variables were reported as means with standard deviations (SDs) for approximately normally distributed data, or medians with interquartile ranges (IQRs) for skewed data, as presented in the original studies. Categorical variables were summarised as proportions with corresponding numerators and denominators. For studies reporting associations with mortality, we extracted effect estimates (odds ratios [ORs], adjusted odds ratios [aORs], hazard ratios [HRs], and adjusted hazard ratios [aHRs]) with 95% confidence intervals (CIs) and *p* values, where available. When effect estimates were not reported, the direction and statistical significance of associations were described as presented in the primary studies. Statistical reporting followed established guidance for clinical research to ensure appropriate presentation of effect estimates, measures of uncertainty, and summary statistics [40]. Data extraction and management were performed using Microsoft Excel 2021 (Microsoft Corporation, Redmond, WA, USA).

## 12 Results

### 12.1 Study Selection and Geographical Distribution

**FIGURE 1:**
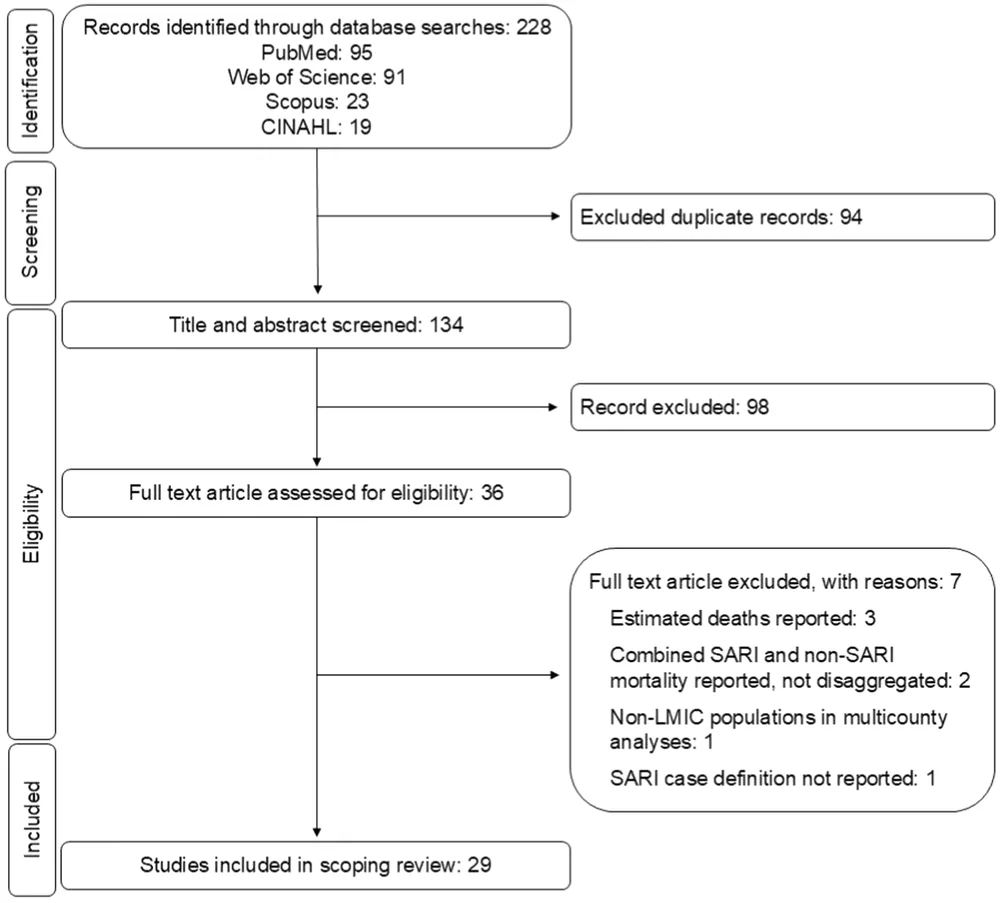
PRISMA flow diagram of the literature search and study selection process, illustrating identification, screening, eligibility assessment, and inclusion of studies reporting mortality among patients with severe acute respiratory infection in low‐ and middle‐income countries.

A total of 228 records were identified through database searches. After removal of duplicates and exclusion of non‐English or non–full‐length articles, 134 records were retained for title and abstract screening. Of these, 98 records were excluded, and 36 articles underwent full‐text assessment for eligibility. Twenty‐nine studies met the inclusion criteria and were included in this scoping review (Figure 1). The included studies were conducted across four regions, with most from South Asia (13 studies) and the Middle East and North Africa (11 studies), followed by sub‐Saharan Africa (SSA) (3 studies) and East Asia and the Pacific (2 studies). Overall, the studies represented 13 LMICs, most frequently India (*n* = 9), Egypt (*n* = 7), and Bangladesh (*n* = 5), followed by Yemen (*n* = 2) and Tunisia (*n* = 2), with one study each from Jordan, Uganda, Viet Nam, Kenya, Mozambique, the Philippines, and Nepal (Table 2 and Supporting Information Table S3).

**TABLE 2:** Characteristics and mortality among patients with severe acute respiratory infection in low‐ and middle‐income countries reported in the reviewed studies.

Author, year, and country	Study design	Focus area of the study	SARI case definition	SARI sample size (*n*)	Age, years (mean or median)	Male sex (%)	Total deaths, *n* (%)	In‐hospital deaths, *n* (%)	Post‐discharge deaths, *n* (%)
Abu Elhassan et al. (2020), Egypt [41]	Prospective surveillance	Clinical outcomes and mortality among SARI patients admitted to the ICU	WHO	3207	Not reported (all ages)	Not reported	24 (11%)	24 (11%)	Not reported
Akhtar et al. (2021), Bangladesh [42]	Hospital‐based descriptive study	SARS‐CoV‐2 and influenza virus coinfection and mortality among SARI patients	WHO	1986	28 years (IQR 1.2–53)	68%	205 (~10%)	106 (5.3%)	99 (5.6%)
Al Amad et al. (2019), Yemen [43]	Retrospective sentinel surveillance analysis	Severity and fatal outcomes of SARI associated with influenza and non‐influenza respiratory viruses	WHO	1811	Not reported (all ages)	49%	145 (8.0%)	145 (8.0%)	Not reported
Al‐Abdallat et al. (2016), Jordan [44]	Retrospective hospital‐based sentinel surveillance	Epidemiology and seasonality of influenza‐associated hospitalisations among SARI cases	WHO	2891	Not reported (all ages)	Not reported	8 (3%)	8 (3%)	Not reported
Ben Khelil et al. (2023), Tunisia [45]	Retrospective descriptive study	Comparison of clinical characteristics and outcomes of severe influenza vs. COVID‐19 among SARI ICU patients	WHO	223	58.95 years (SD 17.89)	55%	68 (30.5%)	68 (30.5%)	Not reported
Boussarsar et al. (2023), Tunisia [7]	Prospective sentinel surveillance	Epidemiology, burden, outcomes, and mortality risk factors of SARI in the post–COVID‐19 period	WHO	164	65 years (IQR 52.25–72)	62%	37 (22.6%)	37 (22.6%)	Not reported
Cummings et al. (2016), Uganda [46]	Prospective hospital‐based sentinel surveillance	Epidemiology, spatiotemporal transmission, and risk factors for influenza‐associated SARI and mortality	WHO	3921	1 year (IQR 0.7–3)	54%	23 (1.6%)	23 (1.6%)	Not reported
Dat et al. (2021), Viet Nam [47]	Multicentre prospective observational study	Burden of SARI in critical care units and capacity/use of diagnostics, treatment, and short‐term mortality	WHO	395	74 years (IQR 58–84)	49%	26 (6.6%)	26 (6.6%)	Not reported
Deval et al. (2024), India [48]	Hospital‐based descriptive study	Detection and molecular characterisation of hMPV among paediatric ARI/SARI cases and associated outcomes	WHO	50	Not reported (children)	Not reported	1 (2%)	1 (2%)	Not reported
Deval et al. (2025), India [49]	Hospital‐based cross‐sectional surveillance	Viral aetiology, seasonality, clinical profile, and outcomes of ARI and SARI among children under 5 years	WHO	332	0.8 years (SD 0.9)	65%	41 (12.3%)	41 (12.3%)	Not reported
El Kholy et al. (2013), Egypt [50]	Prospective cohort study	Risk factors for ICU admission, prolonged length of stay, and mortality among children with RSV‐associated SARI	WHO	240	0.3 years (range 0.07–9.9)	58%	12 (5%)	12 (5%)	Not reported
El Kholy et al. (2014), Egypt [51]	Hospital‐based sentinel surveillance	Risk factors for prolonged hospital stay and outcomes among children with viral SARI	WHO	1046	Not reported (children)	Not reported	21 (5.5%)	21 (5.5%)	Not reported
Hassan et al. (2023), Bangladesh [52]	Prospective hospital‐based surveillance	Characterisation of in‐hospital and post‐discharge SARI deaths and factors associated with mortality before and during the COVID‐19 pandemic	WHO	8216	50 years (IQR 32–60)	62%	785 (~10%)	336 (4.1%)	449 (5.7%)
Hassan et al. (2024), Bangladesh [53]	Retrospective hospital‐based surveillance	Burden and temporal comparison of RSV‐associated deaths among under‐5 SARI patients before and during the COVID‐19 pandemic	WHO	11,493	0.5 years (IQR 0.2–1.0)	66%	225 (2.0%)	225 (2.0%)	Not reported
Hatem et al. (2019), Egypt [54]	Prospective hospital‐based sentinel surveillance	Clinical characteristics, viral aetiology, severe outcomes, and mortality among hospitalised SARI patients	WHO	3207	Not reported (all ages)	Not reported	24 (2.2%)	24 (2.2%)	Not reported
Islam et al. (2024), Bangladesh [16]	Prospective hospital‐based surveillance	Magnitude, timing, and factors associated with in‐hospital and post‐discharge mortality among SARI patients	WHO	23,360	20 years (IQR 1.5–48)	65%	984 (~5%)	351 (1.5%)	633 (3.2%)
Kandeel et al. (2016), Egypt [55]	Prospective hospital‐based sentinel surveillance	Morbidity, mortality, seasonality, and mortality risk factors of influenza‐associated SARI hospitalisations	WHO	17,441	Not reported (all ages)	Not reported	53 (1.8%)	53 (1.8%)	Not reported
Kandeel et al. (2023), Egypt [56]	Multicentre cross‐sectional hospital survey	Viral and bacterial etiologies, clinical characteristics, severity, and in‐hospital outcomes of paediatric SARI	WHO	317	0.7 years (IQR 0.3–2.0)	62%	10 (3.2%)	10 (3.2%)	Not reported
Koul et al. (2019), India [57]	Single‐centre prospective hospital surveillance	Epidemiology, clinical characteristics, mortality, and economic costs of SARI and influenza hospitalisations among adults with diabetes	WHO	192	66 years (range 35–93)	44%	22 (11.4%)	22 (11.4%)	Not reported
Kumar et al. (2022), India [58]	Retrospective hospital‐based observational study	SARS‐CoV‐2 positivity, isolation policy, and in‐hospital outcomes among children with and without SARI	WHO	1155	Not reported (children)	Not reported	17 (34%)	17 (34%)	Not reported
Lucinde et al. (2024), Kenya [59]	Prospective multi‐hospital sentinel surveillance	Description of SARI admissions and outcomes and estimation of COVID‐19 vaccine effectiveness against admission and inpatient mortality	Locally adapted	17,950	50 years (IQR 35–67)	53%	3827 (21.3%)	3827 (21.3%)	Not reported
Meena et al. (2022), India [60]	Retrospective hospital‐based observational study	Comparison of clinical characteristics, severity, and in‐hospital mortality between RT‐PCR–positive and RT‐PCR–negative COVID‐19 SARI patients	WHO	500	53.8 years (SD 15.9)	58%	189 (39.3%)	189 (39.3%)	Not reported
Moorthy et al. (2012), India [61]	Prospective hospital‐based surveillance	Estimation of hospitalisation, ICU admission, and mortality burden during the 2009 pandemic influenza (H1N1) using hospital surveillance data	WHO	1185	Not reported (all ages)	Not reported	138 (11.6%)	138 (11.6%)	Not reported
Pale et al. (2017), Mozambique [62]	Prospective hospital‐based surveillance	Epidemiology, viral aetiology (RSV and influenza), and clinical outcomes, including mortality among children with SARI	WHO	450	0.50 years (IQR 0.2–1.08)	52%	2 (0.4%)	2 (0.4%)	Not reported
Pamaran et al. (2013), Philippines [63]	Enhanced ILI/SARI surveillance study	Epidemiological and clinical characterisation of pandemic and post‐pandemic influenza A(H1N1)pdm09	Locally adapted	98	Not reported (all ages)	Not reported	2 (2.0%)	2 (2.0%)	Not reported
Pannu et al. (2021), India [64]	Prospective hospital‐based cohort	Comparison of aetiology, severity, and outcomes of COVID‐19 vs. non‐COVID‐19 SARI during early pandemic phase	WHO (modified)	212	50.3 years (SD 15.9)	60%	63 (28.9%)	63 (28.9%)	Not reported
Rave et al. (2025), Nepal [18]	Prospective cost‐of‐illness study	Estimation of healthcare system, household, and societal costs of RSV and non‐RSV (S)ARI in young children	WHO	646	Not reported (children)	Not reported	12 (~2%)	8 (1.2%)	4 (0.6%)
Sharma et al. (2021), India [65]	Hospital‐based cross‐sectional study	Comparison of clinical features and mortality between COVID‐19 SARI and non‐COVID‐19 SARI patients	WHO	500	Not reported (18–85 years)	64%	115 (23.0%)	115 (23.0%)	Not reported
Thabet et al. (2016), Yemen [66]	Retrospective hospital‐based SARI surveillance	Aetiology, demographics, seasonality, underlying conditions, and outcomes of SARI hospitalisations	WHO	1346	1.0 year (range 0.5–94.0)	61%	25 (1.9%)	25 (1.9%)	Not reported

Abbreviations: aHR, adjusted hazard ratio; aOR, adjusted odds ratio; ARDS, acute respiratory distress syndrome; CI, confidence interval; hMPV, human metapneumovirus; HR, hazard ratio; ICU, intensive care unit; IQR, interquartile range; OR, odds ratio; qSOFA, quick Sequential Organ Failure Assessment; RSV, respiratory syncytial virus; SARI, severe acute respiratory infection; SD, standard deviation; SpO_2_, peripheral oxygen saturation.

Operational definitions:

SARI case definition: SARI was defined according to the World Health Organization case definition unless otherwise specified.

Post‐discharge mortality proportion was calculated after excluding in‐hospital deaths and patients lost to follow‐up, where reported.

### 12.2 SARI Case Definition and Study Settings

Across the 29 included studies, the WHO SARI case definition was applied in 26 studies (89.7%). Two studies (6.9%) used locally adapted definitions, and one study (3.4%) applied a modified WHO definition (Supporting Information Table S4). All studies were conducted in hospital settings. Most were based on established surveillance platforms (21/29, 72.4%), while the remainder relied on retrospective review of hospital medical records (8/29, 27.6%). SARI enrolment occurred across diverse clinical settings, including general medical and paediatric wards, specialised SARI isolation wards, high‐dependency units, and intensive care units (ICU). Four studies did not specify the exact ward or were conducted across general inpatient settings. Six studies (20.7%) focused specifically on intensive or critical care settings, including ICUs, coronary care units, paediatric ICUs, high‐dependency units, or emergency wards (Supporting Information Table S4).

### 12.3 Study Population Characteristics

The SARI sample sizes across the included studies ranged from 50 to 23,360 patients, with the largest cohort reported from in Bangladesh in pracademic period (*n* = 23,360). Reported age distributions varied markedly by study population; the median age of patients with SARI ranged from 0.3 years (range 0.07–9.9) in paediatric RSV cohorts in Egypt to 74 years (IQR 58–84) in adult critical care cohorts in Viet Nam. The proportion of male patients ranged from 43.8% to 67.6%, with the highest male predominance reported during the first wave of the COVID‐19 pandemic in Bangladesh (Table 2).

### 12.4 Respiratory Pathogen Testing Strategies

Respiratory pathogen testing strategies varied substantially across studies. The number of pathogens tested per study ranged from one to 20, with 13 studies (44.8%) testing a single pathogen, four studies (13.8%) testing two to four pathogens, and 12 studies (41.4%) using multiplex diagnostic panels testing five or more pathogens. Influenza viruses were the most frequently tested pathogens, assessed in 24 of 29 studies (82.8%). SARS‐CoV‐2 was tested in 11 studies (37.9%), predominantly during the COVID‐19 pandemic period. Other respiratory viruses commonly included in testing panels were respiratory syncytial virus, human metapneumovirus, parainfluenza viruses, adenovirus, rhinovirus, enterovirus, human bocavirus, and seasonal coronaviruses. Testing for bacterial respiratory pathogens was uncommon and was reported in only four studies (13.8%), all of which used broader diagnostic panels (Supporting Information Table S4).

### 12.5 Mortality Outcomes and Associated Factors

All included studies reported in‐hospital mortality, with proportions ranging from 0.4% to 39.3%. Four studies additionally reported post‐discharge mortality, accounting for 0.6% to 5.7% of deaths among discharged patients (Table 2). Thirteen studies examined factors associated with mortality: 11 assessed factors associated with in‐hospital mortality, one examined factors associated with post‐discharge mortality, and one reported factors associated with combined in‐hospital and post‐discharge mortality without disaggregation by timing of death (Table 3).

**TABLE 3:** Factors associated with in‐hospital and post‐discharge mortality among patients with severe acute respiratory infection in low‐ and middle‐income countries reported in the reviewed studies.

	Demographic factors		Health‐seeking behaviour	Health‐system factors	Care‐related indicator	Underlying conditions		Clinical presentation & severity markers					Radiological abnormalities	Respiratory pathogen aetiology	
Author, year, and country	Age	Male sex	Delayed hospitalisation	Sentinel site	LOS	Comorbidity	Smoking history	Severe clinical signs	Advanced care requirement	Critical illness complications	Inflammatory blood biomarkers	Severity scores	Chest X‐ray abnormalities	Flu	SARS‐CoV‐2
Factor associated with in‐hospital mortality															
Al Amad et al. (2019), Yemen [43]	Y			Y		Y								Y	
Boussarsar et al. (2023), Tunisia [7]	Y					Y				Y					
Cummings et al. (2016), Uganda [46]	Y	Y													
Dat et al. (2021), Viet Nam [47]										Y		Y			
El Kholy et al. (2013), Egypt [50]						Y			Y						
Kandeel et al. (2016), Egypt [55]	Y		Y			Y								Y	
Lucinde et al. (2024), Kenya [59]		Y				Y		Y			Y				Y
Meena et al. (2022), India [60]						Y		Y			Y				Y
Moorthy et al. (2012), India [61]						Y		Y							
Pannu et al. (2021), India [64]									Y			Y			
Sharma et al. (2021), India [65]	Y										Y		Y		Y
Factor associated with post‐discharge mortality															
Islam et al. (2024), Bangladesh [16]					Y	Y		Y	Y						
Factors associated with mortality (in‐hospital and post‐discharge combined)															
Hassan et al. (2023), Bangladesh [52]						Y	Y	Y							Y

*Note:* Y: factor independently associated with mortality based on multivariable analysis (or univariable analysis if multivariable analysis was not conducted), as reported in the original studies. Blank cells indicate that the factor was not reported, not assessed, or not statistically significant.

Abbreviations: Flu, influenza virus; LOS, length of hospital stay; SARS‐CoV‐2, severe acute respiratory syndrome coronavirus 2.

Operational definitions:

Delayed hospitalisation: delay from symptom onset to admission (≥ 5 days); Sentinel site: paediatric tertiary hospital (Aden site) or general tertiary hospital (Sana'a site); Severe clinical signs: ≥ 1 of difficulty breathing, low peripheral oxygen saturation (SpO_2_), hypothermia (< 360°C), chest in‐drawing, reduced breath sounds, bronchial breathing, or wheeze at hospitalisation; Advanced care requirement: intensive care unit admission and/or advanced ventilatory support; Critical illness complications: acute respiratory distress syndrome and/or early septic shock within 24 h of admission; Inflammatory blood biomarkers: ≥ 1 of high neutrophil–lymphocyte ratio, abnormal neutrophil or lymphocyte percentages, abnormal serum procalcitonin, elevated ferritin or d‐dimer, raised total leucocyte count, anaemia (haemoglobin < 11.5 g/dL), leucocytosis (> 12 × 10^9^/L), or thrombocytopenia (platelet count < 150 × 10^9^/L); Severity scores: early quick Sequential Organ Failure Assessment (qSOFA) ≥ 2 or high Acute Physiology and Chronic Health Evaluation II score.

### 12.6 In‐Hospital Mortality Patterns

Analysis of in‐hospital mortality across the 29 included studies demonstrated substantial heterogeneity, with reported rates ranging from 0.4% (2/450) in a paediatric SARI surveillance study in Mozambique [62] to 39.3% (189/481) among patients hospitalised with COVID‐19–associated SARI in India during the COVID‐19 pandemic [60]. Higher in‐hospital mortality was consistently observed in studies focusing on critically ill or high‐risk populations, including ICU patients with influenza or COVID‐19 in Tunisia (30.5%, 68/223) [45], adults presenting with SARI to emergency medical services in northern India during the early phase of the COVID‐19 pandemic (28.9%, 63/212) [64], adults with diabetes hospitalised with SARI in India (11.4%, 22/192) [57], and paediatric patients with SARS‐CoV‐2 infection in India (34.0%, 17/50) [58]. In contrast, large‐scale hospital‐based SARI surveillance studies reported comparatively lower in‐hospital mortality, including 1.5% (351/23,360) in Bangladesh [16] and 1.8% (53/2936) among influenza‐associated SARI cases in Egypt [55], both conducted before the COVID‐19 pandemic, and 2.0% (225/11,493) in a paediatric respiratory syncytial virus–focused surveillance study conducted before and during the COVID‐19 pandemic in Bangladesh [53] (Table 2).

### 12.7 Factors Associated With In‐Hospital SARI Mortality

Overall, 11 studies reported factors associated with in‐hospital mortality among patients with SARI. Of these, nine used multivariable logistic regression or Cox proportional hazards models, while two reported univariable associations. Reported factors encompassed demographic characteristics, underlying comorbidities, clinical presentation and severity markers (including laboratory abnormalities), pathogen‐related factors, and health‐system factors (Table 3).

## 13 Demographic Factors

Advanced age was associated with increased in‐hospital mortality in multiple studies, though the magnitude varied. In a Tunisian ICU surveillance study during the post‐COVID‐19 period, each additional year of age was associated with a 3% increase in the odds of death (aOR 1.028, 95% CI 1.001–1.056; *p* = 0.04) [7]. In contrast, a Yemeni surveillance study reported an inverse association, with increasing age linked to lower death odds (OR 0.98, 95% CI 0.97–0.99), consistent with higher case fatality among children (< 15 years) than older patients [43]. Ugandan evidence suggested a non‐linear relationship, with adult aged 35–54 years more likely to die in hospital than other age groups [46]. Similarly, an Indian study reported that among SARI died, those with COVID‐19 were significantly older than those without COVID‐19 (*p* < 0.001), though age was not independently associated within the COVID‐19‐positive group (*p* = 0.20), the statistical test for this comparison was not specified [65]. Male sex was associated with higher mortality in studies from East Africa [46, 59]. In Kenya, male had 57% higher odds of death than female (aOR 1.57, 95% CI 1.35–1.82) [59]; similarly, a Ugandan study reported that male sex was associated with increased in‐hospital mortality [46].

### 13.1 Comorbidities and Chronic Conditions

Pre‐existing chronic conditions were consistently associated with in‐hospital mortality. Evidence from Yemen and Egypt showed any chronic disease was an independent factor of death, with substantially elevated odds among Yemeni children (aOR 18.3, 95% CI 6.6–51.2) [43], and a more moderate association across all ages in Egypt (aOR 2.56, 95% CI 1.43–4.58) [55]. In India, chronic kidney disease and autoimmune disease were independently associated with mortality (non‐survivors vs. survivors: 13.3% vs. 1.8%, *p* = 0.03; and 2.4% vs. 0.6%, *p* = 0.04) [60]. Another Indian study reported asthma, haematological malignancy, and transplant status were associated with ICU admission and death, though comorbidity‐specific estimates were not consistently reported [61]. Similarly, in Egyptian children with RSV‐associated SARI, congenital heart disease was independently associated with mortality (OR 18.3, 95% CI 1.35–246.0; *p* = 0.03) [50].

### 13.2 Clinical Presentation, Physiological Severity, and Laboratory Markers

Markers of severe clinical presentation and physiological derangement showed strong and consistent associations with in‐hospital mortality. Low oxygen saturation (SpO_2_ < 90%) was a key factor associated with death Kenya (aOR 1.69, 95% CI 1.38–2.06) [59] and India (mean SpO_2_: 82.3% ± 13.7% in non‐survivors vs. 90.6% ± 9.0% in survivors; *p* < 0.001) [60]. Mechanical ventilation requirement was strongly associated with mortality in Egypt (OR 449.4, 95% CI 23.6–8556) [50] and early ventilator use in non–COVID‐19 cases independently associated with death in India (*p* = 0.04) [64]. Elevated inflammatory markers were significantly associated with death in India: non‐survivors had higher median neutrophil–lymphocyte ratio (16.5 [IQR 8.7–32.5] vs. 8.3 [IQR 4.7–15.9], *p* = 0.04), ferritin (821.2 [IQR 524–1680.8] vs. 578 [IQR 317.8–893.7] ng/mL, *p* = 0.05), and d‐dimer (2.1 [IQR 1.1–6.7] vs. 1.1 [IQR 0.6–1.9] μg/mL, *p* = 0.02) [60]. Thrombocytopenia (OR 32.86, 95% CI 1.29–838.6) [50], neutrophilia, and lymphopenia (median neutrophils 88.2% vs. 77.0%, *p* < 0.001; median lymphocytes 6.7% vs. 15.5%, *p* < 0.001) [65] were also associated with death. A qSOFA score of 2 or higher within 24 h of critical care admission was associated with increased mortality in Viet Nam (aHR 3.41, 95% CI 1.25–9.34) [47], and acute respiratory distress syndrome was strongly associated with mortality in Tunisia (aOR 19.135, 95% CI 5.78–63.31, *p* < 0.001) [7].

### 13.3 Pathogen‐Related Factors

Pathogen‐specific associations with mortality were reported. Infection with influenza B virus was associated with higher fatality odds among Yemeni children aged < 15 years (aOR 6.8, 95% CI 1.9–23.7, *p* = 0.003) [43], whereas influenza A infection was associated with greater mortality risk than influenza B in Egypt (aOR 2.96, 95% CI 1.37–6.40, *p* < 0.05) [55]. SARS‐CoV‐2 positivity at admission and severe COVID‐19 were independently associated with in‐hospital mortality in Kenya (aOR 1.28, 95% CI 1.07–1.52, *p* = 0.008) [59] and severe COVID‐19 was independently associated with mortality among RT‐PCR‐positive patients in India [60].

### 13.4 Health‐Seeking Behaviour

Delayed presentation to hospital was associated with increased in‐hospital mortality. An Egyptian study among influenza‐positive patients found that admission ≥ 5 days after symptom onset had higher death odds than presentation within 2 days (aOR 7.24, 95% CI 3.17–16.57). A duration of 3–4 days from symptom onset to hospitalisation was also associated with increased odds of death, though not statistically significant (aOR 1.61, 95% CI 0.70–3.69) [55].

### 13.5 Health‐System Factor

Health‐system–related factors also influenced in‐hospital mortality. A Yemeni surveillance study found that admission to the Aden site (Paediatric Tertiary Hospital) was associated with higher odds of mortality compared with admission to the Sana'a site (General Tertiary Hospital) (aOR 5.4, 95% CI 2.3–12.5). This association was observed among children under 15 years, whereas no statistically significant association was identified among these aged 15 years and older (aOR 1.3, 95% CI 0.15–11.5) [43].

### 13.6 Cause of In‐Hospital Death

Although several included studies examined factors associated with in‐hospital mortality, only one study reported pathogen‐specific causes of in‐hospital death among children with SARI [56]. Regarding cause‐of‐death ascertainment, no study used formal MCCD or verbal autopsy. The Egyptian study reporting causes of death relied on physician‐assigned diagnoses and laboratory confirmation but did not report clinically adjudicated underlying causes (e.g., respiratory failure and sepsis) [56]. The study population comprised hospitalised children with SARI (median age: 0.7 years, IQR 0.32–2.03). Respiratory specimens were tested for a broad panel of pathogens, including influenza viruses, RSV, rhinovirus, adenovirus, human metapneumovirus, SARS‐CoV‐2, Haemophilus influenzae (non–type B), and Streptococcus pneumoniae using real‐time reverse transcription polymerase chain reaction (rRT‐PCR). Rhinovirus was reported as the most common cause of in‐hospital deaths (8.7%, 2/23), followed by RSV (1.4%, 2/146). The study further reported that viral–bacterial co‐infections were associated with substantially higher rates of severe outcomes compared with viral‐only or bacterial‐only infections [56].

### 13.7 Post‐Discharge Mortality

Post‐discharge mortality was reported in only four included studies, all conducted in South Asia, with three from Bangladesh and one from Nepal. Reported post‐discharge mortality ranged from 0.6% (4/638) in a paediatric cost‐of‐illness study from Nepal in 2023 [18] to 5.7% (449/7780) among adults during the pre‐ and COVID‐19 pandemic periods in a surveillance study from Bangladesh [52]. Two additional reports from the same Bangladeshi surveillance platform documented post‐discharge mortality rates of 5.6% (99/1766) during the first COVID‐19 wave [42] and 3.2% (633/20,044) in the pre‐pandemic period, where post‐discharge deaths accounting for the majority of total reported mortality [16].

### 13.8 Factors Associated With Post‐Discharge SARI Mortality

Only one of the four studies reporting post‐discharge mortality evaluated factors associated with post‐discharge death [16]. This study identified distinct risk profiles by age group. Among children (< 18 years), post‐discharge mortality was associated with difficulty breathing (aHR 1.84, 95% CI 1.12–3.01, *p* = 0.02), longer hospital stay (aHR 1.09 per day, 95% CI 1.06–1.12, *p* < 0.001), and underlying heart disease (aHR 8.53, 95% CI 3.16–23.08, *p* < 0.001). Among adults (≥ 18 years), elevated risk of post‐discharge death was associated with difficulty breathing (aHR 2.28, 95% CI 1.73–2.99, *p* < 0.001), COPD (aHR 1.71, 95% CI 1.35–2.16, *p* < 0.001), and admission to the ICU during the index hospitalisation (aHR 5.19, 95% CI 1.92–13.99, *p* < 0.001) (Table 3) [16].

### 13.9 Cause of Post‐Discharge Death

None of the included studies reported definitive causes of death following hospital discharge among patients with SARI. Three Bangladeshi studies assessed post‐discharge mortality but did not specify underlying causes of death [16, 42, 52]. Similarly, the Nepalese study reported post‐discharge deaths without specifying causes [18].

### 13.10 Factors Associated With Mortality (In‐Hospital and Post‐Discharge Combined)

One study from Bangladesh reported in‐hospital and post‐discharge deaths separately but analysed mortality as a combined outcome among adults with SARI during the pre‐pandemic and pandemic periods [52]. During the pre‐pandemic period, smoking history (aHR 1.41, 95% CI 1.09–1.83, *p* = 0.01) and having ≥ 1 comorbid condition (aHR 1.33, 95% CI 1.01–1.73, *p* = 0.04) were independently associated with mortality. Respiratory difficulty at admission was associated with increased mortality in both the pre‐pandemic (aHR 2.36, 95% CI 1.65–3.36, *p* < 0.001) and pandemic periods (aHR 2.30, 95% CI 1.57–3.35, *p* < 0.001) (Table 3) [52].

## 14 Discussion

This scoping review synthesises evidence on factors contributing to both in‐hospital and post‐discharge mortality among patients hospitalised with SARI in LMICs. Three key observations emerged. First, the SARI mortality literature dominated by in‐hospital outcomes, with post‐discharge mortality rarely reported. Second, in‐hospital mortality is consistently associated with older age, male sex, underlying comorbidities, markers of severe disease at presentation, pathogen‐specific effects, and delayed access to care, though cause‐of‐death data are scarce. Third, where assessed, post‐discharge deaths account for a substantive proportion of overall mortality, yet contributing factors and causes remain poorly characterised.

Consistent with the global literature on respiratory viral infections, reported in‐hospital mortality among patients with SARI varied widely across studies, ranging from less than 1% in paediatric surveillance cohorts to nearly 40% among critically ill adults with SARS‐CoV‐2–associated SARI during the early COVID‐19 pandemic [60, 62, 67, 68]. This heterogeneity likely reflects differences in patient populations, circulating pathogens, healthcare settings, and illness severity. ICUs or emergency setting studies reported higher mortality [7, 45, 64], whereas large surveillance studies from Bangladesh and Egypt reported lower fatality proportions due to broader case ascertainment and inclusion of less severe disease [16, 55]. Conversely, studies restricted to high‐risk populations, including patients with comorbidities, critical illness, or COVID‐19, demonstrated substantially higher mortality [45, 60]. Thus, SARI mortality estimates should be interpreted in the context of study design and care setting.

The factors associated with in‐hospital mortality identified in this review, including older age, male sex, underlying comorbidities, severe clinical presentation, and pathogen‐specific effects, are well established in the global literature on severe respiratory infections [16, 69, 70]. Indicators of clinical severity (hypoxaemia, mechanical ventilation, development of acute respiratory distress syndrome, and elevated inflammatory biomarkers) were consistently associated with increased mortality risk, highlighting the contribution of advanced physiological compromise to fatal outcomes [47, 59, 60]. Pathogen‐specific differences, particularly the higher mortality associated with SARS‐CoV‐2, likely reflect a combination of intrinsic viral virulence, host vulnerability, and contextual factors related to epidemic phase and healthcare system strain [59, 60].

Health‐seeking behaviour contributed to SARI mortality. Delayed presentation to hospital was reported to be associated with an increased risk of death [55], likely reflecting barriers to timely care‐seeking. Although the included studies did not examine reasons for delay, previous research implicates financial constraints, sociocultural norms, and healthcare access and healthcare accessibility at individual, household, and community levels [71]. In LMICs, where timely access to care is often constrained, delayed care‐seeking represents a potentially modifiable vulnerability requiring further investigation.

We found that health‐system factors emerged as important contributors to mortality, with variation across settings. Higher mortality in the paediatric tertiary hospital compared with the general tertiary hospital [43] likely reflects a combination of differences in case mix, referral pathways, or service capacity. Persistent shortages of trained staff, essential medicines, oxygen, diagnostic infrastructure, and heterogeneity in laboratory testing practices across many LMICs may further compromise patient outcomes [72, 73, 74]. Limited molecular testing may also lead to under‐detection of pathogens [75, 76]. Constraints in hospital bed capacity and restrictive admission practices may result in hospitalisation of only the most severe cases, potentially inflating in‐hospital mortality while underestimating the overall burden of SARI [16, 41, 74, 77, 78]. Together, these findings highlight the importance of interpreting SARI mortality in the context of health‐system capacity and diagnostic availability in LMICs [1, 74, 78, 79].

A major evidence gap identified in this review is the paucity of cause‐of‐death data and the absence of standardised ascertainment methods. None of the included studies used formal MCCD or verbal autopsy. The only study reporting in‐hospital causes of death relied on physician‐assigned diagnoses and laboratory‐confirmed pathogen identification, reporting pathogen‐specific attribution rather than clinically adjudicated underlying causes such as respiratory failure, sepsis, or multi‐organ dysfunction [56]. No study reported causes of post‐discharge mortality. The absence of standardised cause‐of‐death assessment, particularly for deaths occurring outside healthcare facilities, limits understanding of whether mortality resulted from unresolved infection, secondary complications, critical illness sequelae, or worsening comorbidities. In contrast, studies from high‐income settings have shown that post‐discharge mortality is frequently associated with cardiovascular complications and recurrent infections [80, 81, 82]. Future research in LMICs would benefit from prioritising prospective longitudinal designs incorporating standardised cause‐of‐death assessment, including verbal autopsy for deaths occurring outside healthcare facilities where MCCD is unavailable, alongside active post‐discharge follow‐up.

An important finding of this review is the marked underrepresentation of SSA, which contributed only three of 29 included studies despite bearing a substantial burden of respiratory infections and related mortality. This limited representation restricts the generalisability of findings and highlights a major evidence gap [1]. Possible reasons include competing health priorities, limited diagnostic capacity, fragmented surveillance systems, and insufficient research funding in many SSA countries [83, 84]. The lack of robust SARI mortality data is particularly concerning given the high prevalence of HIV, tuberculosis, and malnutrition in the region [85, 86, 87, 88]. Strengthening sentinel surveillance systems, integrating SARI monitoring into existing platforms, and supporting post‐discharge follow‐up studies are warranted to address this gap [16, 89].

Strengthening surveillance and diagnostic capacity for SARI in LMICs requires a combination of scalable, short‐term solutions and longer‐term system investments. Rapid diagnostic tests (RDTs) for pathogens such as influenza, RSV, and SARS‐CoV‐2 can improve frontline diagnosis and support timely clinical management where laboratory capacity is limited [89, 90]. However, advanced laboratory infrastructure, including molecular testing and multiplex rRT‐PCR, remains essential for confirmatory diagnosis and surveillance of emerging threats [91]. A tiered diagnostic approach combining RDTs at primary facilities with molecular confirmation at higher‐level laboratories may provide a cost‐effective model for LMICs [92, 93]. In addition, strengthening digital reporting systems and integrating SARI surveillance into existing influenza and COVID‐19 platforms could further improve surveillance capacity and reduce preventable mortality [94, 95, 96].

The findings of this review highlight several priority areas for policy action to accurately assess and reduce SARI‐associated mortality in LMICs. First, given that post‐discharge mortality accounted for a substantial proportion of reported deaths, SARI surveillance systems should be strengthened to routinely capture both in‐hospital and post‐discharge mortality, enabling a more complete assessment of disease burden and patient outcomes [16]. Second, the finding that nearly half of included studies tested only a single pathogen and that few studies included bacterial testing, despite evidence that co‐infections increase mortality, supports expanding multiplex molecular diagnostic capacity in LMIC hospitals to improve detection of both viral and bacterial aetiologies and inform clinical management [91]. Third, the complete absence of standardised cause‐of‐death reporting, with no study using MCCD or verbal autopsy, highlights the need to integrate cause‐of‐death ascertainment into existing surveillance systems and healthcare services to improve understanding of mortality patterns and inform public health policy [37]. Finally, strengthening in‐hospital and post‐discharge care should be a priority to improve patient outcomes and reduce preventable SARI‐associated mortality [16].

This review has several strengths, including its explicit focus on both in‐hospital and post‐discharge mortality among patients with SARI in LMICs, providing a more comprehensive perspective on disease burden. Methodological rigour was ensured through adherence to the Arksey and O'Malley framework and PRISMA‐ScR guidelines. The search spanned over 25 years across four major databases and captured diverse LMIC settings and multiple epidemic periods, thereby enhancing the relevance and generalisability of the findings.

However, several limitations should be noted. The included studies were heterogeneous in design, populations, pathogens, and clinical settings, limiting comparability and the feasibility of quantitative synthesis. Second, evidence was predominantly restricted to in‐hospital mortality, with relatively few studies reporting post‐discharge deaths, associated factors, or causes of death, resulting in an incomplete characterisation of SARI‐related mortality. Third, cause‐of‐death data were not standardised and were based on physician‐assigned clinical diagnoses from hospital medical records or pathogen detection, as no study used formal MCCD or verbal autopsy, potentially leading to misclassification. Fourth, the geographic concentration of studies in South Asia and North Africa limits generalisability to all LMIC regions, particularly SSA. Fifth, reliance on hospital‐based surveillance may also underestimate the true burden of SARI mortality by excluding community deaths. Sixth, bacterial testing was reported in only a minority of studies, despite evidence that bacterial co‐infection and secondary pneumonia increase mortality in influenza and severe COVID‐19 [97, 98], this likely understates bacterial contributions to SARI mortality. Seventh, restricting inclusion to English‐language publications or those with an available English translation may have introduced language bias and excluded relevant research published in other languages. Finally, consistent with scoping review methodology, study quality was not formally assessed.

## 15 Conclusion

This scoping review indicates that evidence on SARI‐associated mortality in LMICs is largely confined to in‐hospital outcomes, with limited assessment of post‐discharge mortality or causes of death. In‐hospital mortality is consistently associated with older age, underlying comorbidities, severe clinical presentation, and delayed access to care, reflecting persistent health‐system vulnerabilities. Standardised post‐discharge follow‐up and systematic cause‐of‐death ascertainment are needed to improve clinical management, strengthen surveillance, and inform targeted interventions to reduce SARI‐associated mortality in resource‐limited settings.

## Author Contributions

**Md Ariful Islam:** conceptualisation, methodology, software, resources, validation, formal analysis, data curation, writing – original draft, visualisation, writing – review and editing, project administration, investigation. **Holly Seale:** conceptualisation, methodology, validation, writing – original draft, supervision, writing – review and editing. **Md Saiful Islam:** conceptualisation, methodology, validation, writing – review and editing, writing – original draft, supervision. **Saju Bhuiya:** software, resources, formal analysis, data curation, visualisation, writing – review and editing, investigation. **Md Zakiul Hassan:** writing – review and editing. **Quazi Fayeza Sultana:** software, resources, data curation, writing – review and editing, investigation. **Fahmida Chowdhury:** writing – review and editing. **Abrar Ahmad Chughtai:** conceptualisation, methodology, software, validation, writing – original draft, supervision, writing – review and editing, project administration.

## Funding

The authors have nothing to report.

## Ethics Statement

The authors have nothing to report.

## Conflicts of Interest

The authors declare no conflicts of interest.

## Transparency Statement

Md Ariful Islam affirms that this manuscript is an honest, accurate, and transparent account of the study being reported; that no important aspects of the study have been omitted; and that any discrepancies from the study as planned (and, if relevant, registered) have been explained.

## Supporting information


Supporting File 1

Supporting File 2

Supporting File 3

Supporting File 4

## Data Availability

The data that support the findings of this study are available from the corresponding author upon reasonable request.
