# Supplementary material for: Causes and Contributing Factors of In‐Hospital and Post‐Discharge Mortality Among Patients With Severe Acute Respiratory Infection in Low‐ and Middle‐Income Countries: A Scoping Review
Source: Health Sci Rep. 2026 Sep 13;9(9):e72927. doi: 10.1002/hsr2.72927 (PMC13572787; doi:10.1002/hsr2.72927)
Supplement: Supplementary file 1 [file HSR2-9-e72927-s001.docx]

**Supplementary Table S3** Geographical distribution of included studies reporting severe acute respiratory infection–related mortality in low- and middle-income countries

| **Region** | **Country** | **Number of studies** |
| --- | --- | --- |
| South Asia | India | 8 |
|  | Bangladesh | 4 |
|  | Nepal | 1 |
|  | **Subtotal** | **13** |
| Middle East and North Africa | Egypt | 6 |
|  | Yemen | 2 |
|  | Tunisia | 2 |
|  | Jordan | 1 |
|  | **Subtotal** | **11** |
| Sub-Saharan Africa | Kenya | 1 |
|  | Uganda | 1 |
|  | Mozambique | 1 |
|  | **Subtotal** | **3** |
| East Asia and Pacific | Philippines | 1 |
|  | Viet Nam | 1 |
|  | **Subtotal** | **2** |
| **Total** |  | **29** |
