# Supplementary material for: Causes and Contributing Factors of In‐Hospital and Post‐Discharge Mortality Among Patients With Severe Acute Respiratory Infection in Low‐ and Middle‐Income Countries: A Scoping Review
Source: Health Sci Rep. 2026 Sep 13;9(9):e72927. doi: 10.1002/hsr2.72927 (PMC13572787; doi:10.1002/hsr2.72927)
Supplement: Supplementary file 2 [file HSR2-9-e72927-s002.docx]

**Supplementary Table S2** Chronology of severe acute respiratory infection case definition of World Health Organization, 2005–2025

| Timeline | SARI Case definition | Change [29] | Rationale [29] |
| --- | --- | --- | --- |
| 2005 | **For patients > 5 years:** Sudden onset of fever of ≥38°C and cough or sore throat in the absence of another diagnosis) and shortness of breath or difficulty in breathing and requiring hospital admission [25]. |  |  |
|  | **For patients < 5 years:** in some areas, the IMCI* case definition for pneumonia and severe pneumonia, which is any child aged 2 months to 5 years with cough or difficulty in breathing and breathing faster than 40 breaths/min (ages 1–5 years) or breathing faster than 50 breaths/min (ages 2–12 months); or  the IMCI* case definition for severe pneumonia in any child aged 2 months to 5 years with cough or difficult breathing and unable to drink or breastfeed, or vomits everything, or convulsions, or lethargic or unconscious, or chest indrawing or stridor in a calm child. and requires hospital admission [25]. |  |  |
| 2011 | **For all age groups:** An acute respiratory illness with onset during the previous 7 days requiring overnight hospitalisation that includes: • history of fever or measured fever of ≥ 38°C, AND • cough, AND • shortness of breath or difficulty breathing [26]. | Change ‘sudden onset of fever’ to ‘acute respiratory illness’ | Encompasses a broad, well-known diagnosis. |
|  |  |  | Relates definition to a clinically recognized condition |
|  |  |  | Simpler |
|  |  | Fever > 38°C changed to “history of fever or measured fever of ≥ 38°C | Many patients have taken antipyretics. |
|  |  |  | Fever may be absent in older adults, especially those who are chronically ill. |
|  |  |  | Data from Guatemala and Kenya indicate an improvement in sensitivity. |
|  |  |  | Many sites do not see enough cases to test because of the stringent criterion of measured fever, especially in sub-Saharan Africa, where temperature is unlikely to be measured. |
|  |  | Add a timeframe, of 7 days from symptom onset | New illness superimposed on old must be distinguished. |
|  |  |  | May need to clarify meaning in footnotes or use time parameter |
|  |  | Dropped ‘sore throat’ | No association with influenza in cases of respiratory disease |
|  |  |  | Impossible to assess in infants |
|  |  | The IMCI case definitions for pneumonia and severe pneumonia have been dropped for children < age 5 years.  There is a single SARI case definition for all age groups. | Use of a single SARI case definition for all age groups simplifies implementation. The IMCI case definitions represent a clinical management tool and not very suitable for surveillance. |
| 2014 | **For all age groups:** An acute respiratory infection with history of fever or measured fever of ≥ 38°C and cough; with onset within the last 10 days and requires hospitalisation [28]. | Delete ‘shortness of breath’ and ‘difficulty in breathing’ | These symptoms are not sufficiently specific, do not add to the accuracy of the definition and are often misinterpreted. |
|  |  | • Change onset during ‘7 days’ to ‘10 days’ | SARI cases that present between seven and 10 days after symptom onset appear no less likely to be laboratory-test-positive for influenza as those that present closer to symptom onset. Increasing the time frame to 10 days should therefore allow more cases to be captured, without markedly increasing the use of resources. |

**Abbreviations:** IMCI, Integrated Management of Childhood Illness; SARI, severe acute respiratory infection.
