# Supplementary material for: Causes and Contributing Factors of In‐Hospital and Post‐Discharge Mortality Among Patients With Severe Acute Respiratory Infection in Low‐ and Middle‐Income Countries: A Scoping Review
Source: Health Sci Rep. 2026 Sep 13;9(9):e72927. doi: 10.1002/hsr2.72927 (PMC13572787; doi:10.1002/hsr2.72927)
Supplement: Supplementary file 3 [file HSR2-9-e72927-s003.docx]

**Supplementary Table S4** Pathogens tested in studies reporting severe acute respiratory infection–related mortality in low- and middle-income countries, as reported in the reviewed studies

| **Author, year, country** | **Data source** | **SARI enrolment at hospital (ward/unit)** | **No. of pathogens tested** | **Respiratory viruses tested** | **Bacterial pathogens tested** |
| --- | --- | --- | --- | --- | --- |
| Abu Elhassan et al. (2020), Egypt [69] | Surveillance | ICU | 10 | Influenza viruses; RSV; hMPV; parainfluenza viruses; adenovirus; human bocavirus; enterovirus; rhinovirus | Mycoplasma pneumoniae; Chlamydia pneumoniae |
| Akhtar et al. (2021), Bangladesh [58] | Surveillance | Medicine, paediatrics, CCU, and specialised isolation wards | 2 | Influenza viruses; SARS-CoV-2 | Not tested |
| Al Amad et al. (2019), Yemen [49] | Surveillance | Tertiary hospitals (wards not specified) | 5 | Influenza viruses; RSV; adenovirus; parainfluenza viruses; hMPV | Not tested |
| Al-Abdallat et al. (2016), Jordan [92] | Surveillance | General hospitals (wards not specified) | 1 | Influenza viruses | Not tested |
| Ben Khelil et al. (2023), Tunisia [43] | Surveillance | Medical ICU | 9 | SARS-CoV-2; influenza viruses; RSV; hMPV; adenovirus; human bocavirus; parainfluenza viruses; enterovirus; rhinovirus | Not tested |
| Boussarsar et al. (2023), Tunisia [7] | Surveillance | Medical ICU | 10 | Influenza viruses; RSV; adenovirus; human bocavirus; hMPV; enterovirus; rhinovirus; parainfluenza viruses; seasonal coronaviruses | Mycoplasma pneumoniae |
| Cummings et al. (2016), Uganda [50] | Surveillance | Hospital wards (not specified) | 1 | Influenza viruses | Not tested |
| Dat et al. (2021), Viet Nam [55] | Hospital records | Critical care units (CCUs) | 1 | Influenza viruses | Not tested |
| Deval et al. (2024), India [93] | Hospital records | Paediatric wards and ICU | 7 | hMPV; influenza viruses; SARS-CoV-2; RSV; parainfluenza viruses; adenovirus; rhinovirus | Not tested |
| Deval et al. (2025), India [94] | Surveillance | PICU, HDU, and emergency ward | 7 | Influenza viruses; SARS-CoV-2; RSV; parainfluenza viruses; adenovirus; hMPV; rhinovirus | Not tested |
| El Kholy et al. (2013), Egypt [54] | Hospital records | Paediatric wards and PICU | 5 | RSV; adenovirus; parainfluenza viruses; influenza viruses; hMPV | Not tested |
| El Kholy et al. (2014), Egypt [95] | Surveillance | Paediatric wards and PICU | 5 | RSV; adenovirus; parainfluenza viruses; hMPV; influenza viruses | Not tested |
| Hassan et al. (2023), Bangladesh [57] | Surveillance | Medicine wards, CCU, and COVID-19 isolation wards | 2 | Influenza viruses; SARS-CoV-2 | Not tested |
| Hassan et al. (2024), Bangladesh [48] | Surveillance | Medicine, paediatrics, CCU, and COVID-19 isolation wards | 6 | RSV; influenza viruses; hMPV; adenovirus; parainfluenza viruses; SARS-CoV-2 | Not tested |
| Hatem et al. (2019), Egypt [96] | Surveillance | Medical wards and ICU | 13 | Influenza viruses; RSV; hMPV; parainfluenza viruses; adenovirus; human bocavirus; seasonal coronaviruses; enterovirus; rhinovirus | Mycoplasma pneumoniae; Chlamydia pneumoniae; Legionella pneumophila |
| Islam et al. (2024), Bangladesh [16] | Surveillance | Medicine and paediatric wards; ICU/CCU during part of study | 1 | Influenza viruses | Not tested |
| Kandeel et al. (2016), Egypt [47] | Surveillance | Medical and paediatric inpatient wards | 1 | Influenza viruses | Not tested |
| Kandeel et al. (2023), Egypt [56] | Hospital records | Inpatient wards, intermediate care units, NICU | 20 | Influenza viruses; RSV; parainfluenza viruses; hMPV; rhinovirus; enterovirus; parechovirus; coronaviruses; picornaviruses | Bordetella spp.; Chlamydophila pneumoniae; Haemophilus influenzae (non-type b); Klebsiella pneumoniae; Legionella pneumophila/longbeachae; Moraxella catarrhalis; Mycoplasma pneumoniae; Pneumocystis jirovecii; Salmonella spp.; Staphylococcus aureus; Streptococcus pneumoniae |
| Koul et al. (2019), India [45] | Surveillance | Emergency medicine, internal medicine, endocrinology, pulmonary wards | 1 | Influenza viruses | Not tested |
| Kumar et al. (2022), India [46] | Hospital records | Dedicated paediatric SARI isolation ward | 1 | SARS-CoV-2 | Not tested |
| Lucinde et al. (2024), Kenya [52] | Surveillance | Adult medical wards | 1 | SARS-CoV-2 | Not tested |
| Meena et al. (2022), India [42] | Hospital records | Dedicated adult SARI ward | 1 | SARS-CoV-2 | Not tested |
| Moorthy et al. (2012), India [53] | Surveillance | Medical and paediatric wards | 1 | Influenza viruses | Not tested |
| Pale et al. (2017), Mozambique [41] | Surveillance | Paediatric breastfeeding and respiratory wards | 2 | RSV; influenza viruses | Not tested |
| Pamaran et al. (2013), Philippines [97] | Surveillance | Inpatient wards (not specified) | 1 | Influenza viruses | Not tested |
| Pannu et al. (2021), India [44] | Surveillance | Adult medical emergency SARI isolation ward | 2 | Influenza viruses; SARS-CoV-2 | Not tested |
| Rave et al. (2025), Nepal [18] | Hospital records* | Paediatric wards, HDU, paediatric/neonatal ICU | 1 | RSV | Not tested |
| Sharma et al. (2021), India [51] | Hospital records | COVID-19 suspect ICU | 1 | SARS-CoV-2 | Not tested |
| Thabet et al. (2016), Yemen [98] | Surveillance | Paediatric and internal medicine wards | 5 | Influenza viruses; RSV; adenovirus; hMPV; parainfluenza viruses | Not tested |

Abbreviations:

SARI, severe acute respiratory infection; RSV, respiratory syncytial virus; hMPV, human metapneumovirus; ICU, intensive care unit; CCU, coronary care unit; PICU, paediatric intensive care unit; HDU, high-dependency unit; NICU, neonatal intensive care unit; SARS-CoV-2, severe acute respiratory syndrome coronavirus 2.

Notes:

Pathogens are listed as reported in the primary studies; Not tested indicates that testing was not performed or not reported.

Influenza type and subtype were considered a single pathogen.

Seasonal coronaviruses include 229E, HKU1, NL63, and OC43.

Parainfluenza viruses include types 1–4.

Hospital records* indicates that, in addition to hospital medical records, caregiver interviews were conducted to collect direct medical, direct non-medical, and indirect cost data to estimate total per-patient costs, in accordance with the study objectives.
