# Supplementary material for: Causes and Contributing Factors of In‐Hospital and Post‐Discharge Mortality Among Patients With Severe Acute Respiratory Infection in Low‐ and Middle‐Income Countries: A Scoping Review
Source: Health Sci Rep. 2026 Sep 13;9(9):e72927. doi: 10.1002/hsr2.72927 (PMC13572787; doi:10.1002/hsr2.72927)
Supplement: Supplementary file 4 [file HSR2-9-e72927-s004.docx]

**Supplementary Table S1** Database-specific search strategies for studies of severe acute respiratory infection–related mortality in low- and middle-income countries

| **Database** | **Search strategy** |
| --- | --- |
| **PubMed** | ("severe acute respiratory infection*"[All Fields] AND ( "death*"[All Fields] OR ("Mortality"[MeSH Terms] OR "Mortality"[All Fields] OR "mortalities"[All Fields] OR "Mortality"[MeSH Subheading]) OR ("fatal outcome"[MeSH Terms] OR ("fatal"[All Fields] AND "outcome"[All Fields]) OR "fatal outcome"[All Fields]) OR "Death"[MeSH Terms] OR "Mortality"[MeSH Terms] ) AND ( "Developing Countries"[MeSH Terms] OR "low and middle income countr*"[All Fields] OR "lmic*"[All Fields] OR "low income countr*"[All Fields] OR "middle income countr*"[All Fields] OR "lower-middle income economies"[All Fields] OR "underdeveloped countr*"[All Fields] OR "resource limited setting*"[All Fields] OR ( ( "Afghanistan"[MeSH Terms] OR "Afghanistan"[All Fields] OR "North Korea"[All Fields] OR "South Sudan"[All Fields] OR "Burkina Faso"[All Fields] OR ("Liberia"[MeSH Terms] OR "Liberia"[All Fields]) OR ("Sudan"[MeSH Terms] OR "Sudan"[All Fields] OR "Sudans"[All Fields]) OR ("Burundi"[MeSH Terms] OR "Burundi"[All Fields]) OR ("Madagascar"[MeSH Terms] OR "Madagascar"[All Fields]) OR "Syrian Arab Republic"[All Fields] OR "Central African Republic"[All Fields] OR ("Malawi"[MeSH Terms] OR "Malawi"[All Fields]) OR ("Togo"[MeSH Terms] OR "Togo"[All Fields]) OR ("Chad"[MeSH Terms] OR "Chad"[All Fields]) OR ("Mali"[MeSH Terms] OR "Mali"[All Fields]) OR ("Uganda"[MeSH Terms] OR "Uganda"[All Fields]) OR "Democratic Republic of the Congo"[All Fields] OR ("Mozambique"[MeSH Terms] OR "Mozambique"[All Fields]) OR "Yemen Rep"[All Fields] OR ("Eritrea"[MeSH Terms] OR "Eritrea"[All Fields]) OR ("Niger"[MeSH Terms] OR "Niger"[All Fields]) OR ("Ethiopia"[MeSH Terms] OR "Ethiopia"[All Fields]) OR ("Rwanda"[MeSH Terms] OR "Rwanda"[All Fields]) OR "Gambia"[All Fields] OR "Sierra Leone"[All Fields] OR "Guinea-Bissau"[All Fields] OR ("Somalia"[MeSH Terms] OR "Somalia"[All Fields]) OR ("Angola"[MeSH Terms] OR "Angola"[All Fields]) OR ("Honduras"[MeSH Terms] OR "Honduras"[All Fields]) ) AND "Papua New Guinea"[All Fields] ) OR ("Bangladesh"[MeSH Terms] OR "Bangladesh"[All Fields]) OR ("India"[MeSH Terms] OR "India"[All Fields] OR "Indias"[All Fields]) OR ("Philippines"[MeSH Terms] OR "Philippines"[All Fields] OR "Philippine"[All Fields]) OR ("Benin"[MeSH Terms] OR "Benin"[All Fields]) OR ("Jordan"[MeSH Terms] OR "Jordan"[All Fields]) OR ("Samoa"[MeSH Terms] OR "Samoa"[All Fields] OR "Samoas"[All Fields]) OR ("Bhutan"[MeSH Terms] OR "Bhutan"[All Fields]) OR ("Kenya"[MeSH Terms] OR "Kenya"[All Fields]) OR "Sao Tome and Principe"[All Fields] OR ("Bolivia"[MeSH Terms] OR "Bolivia"[All Fields]) OR ("Kiribati"[MeSH Terms] OR "Kiribati"[All Fields]) OR ("Senegal"[MeSH Terms] OR "Senegal"[All Fields]) OR "Cabo Verde"[All Fields] OR "Kyrgyz Republic"[All Fields] OR "Solomon Islands"[All Fields] OR ("Cambodia"[MeSH Terms] OR "Cambodia"[All Fields]) OR "Lao PDR"[All Fields] OR "Sri Lanka"[All Fields] OR ("Cameroon"[MeSH Terms] OR "Cameroon"[All Fields] OR "Cameroons"[All Fields]) OR ("Lebanon"[MeSH Terms] OR "Lebanon"[All Fields]) OR ("Tajikistan"[MeSH Terms] OR "Tajikistan"[All Fields]) OR ("Comoros"[MeSH Terms] OR "Comoros"[All Fields] OR "Comoro"[All Fields]) OR ("Lesotho"[MeSH Terms] OR "Lesotho"[All Fields]) OR ("Tanzania"[MeSH Terms] OR "Tanzania"[All Fields]) OR "Republic of the Congo"[All Fields] OR ("Mauritania"[MeSH Terms] OR "Mauritania"[All Fields]) OR "Timor-Leste"[All Fields] OR "Cote d'Ivoire"[All Fields] OR "Federated States of Micronesia"[All Fields] OR ("Tunisia"[MeSH Terms] OR "Tunisia"[All Fields]) OR ("Djibouti"[MeSH Terms] OR "Djibouti"[All Fields]) OR ("Morocco"[MeSH Terms] OR "Morocco"[All Fields]) OR ("Uzbekistan"[MeSH Terms] OR "Uzbekistan"[All Fields]) OR "Egypt"[All Fields] OR ("Myanmar"[MeSH Terms] OR "Myanmar"[All Fields] OR "Myanmars"[All Fields]) OR ("Vanuatu"[MeSH Terms] OR "Vanuatu"[All Fields]) OR ("Eswatini"[MeSH Terms] OR "Eswatini"[All Fields]) OR ("Nepal"[MeSH Terms] OR "Nepal"[All Fields]) OR ("Vietnam"[MeSH Terms] OR "Vietnam"[All Fields]) OR ("Ghana"[MeSH Terms] OR "Ghana"[All Fields]) OR ("Nicaragua"[MeSH Terms] OR "Nicaragua"[All Fields]) OR "West Bank and Gaza"[All Fields] OR ("Guinea"[MeSH Terms] OR "Guinea"[All Fields] OR "Guineas"[All Fields]) OR ("Nigeria"[MeSH Terms] OR "Nigeria"[All Fields]) OR ("Zambia"[MeSH Terms] OR "Zambia"[All Fields]) OR ("Haiti"[MeSH Terms] OR "Haiti"[All Fields]) OR ("Pakistan"[MeSH Terms] OR "Pakistan"[All Fields]) OR ("Zimbabwe"[MeSH Terms] OR "Zimbabwe"[All Fields]) ) ) AND (2000/01/01:2025/10/30[pdat]) |
| **Scopus and**  **Web of Science** | ("severe acute respiratory infection*") AND (death* OR “fatal outcome*” OR mortalit*) AND ("resource-limited setting*" OR “resource limited setting*” OR "underdeveloped countr*"OR “under developed countr*” OR "lower-middle income econom*" OR "middle-income countr*" OR "lower middle income econom*" OR "middle income countr*" OR "low-income countr*" OR "low income countr*" OR LMIC OR "low- and middle-income countr*" OR "low and middle income countr*" OR “developing countr*” OR Afghanistan OR "North korea" OR "South Sudan" OR "Burkina Faso" OR Liberia OR Sudan OR Burundi OR Madagascar OR "Syrian Arab Republic" OR "Central African Republic" OR Malawi OR Togo OR Chad OR Mali OR Uganda OR "Democratic Republic of the Congo" OR Mozambique OR "Yemen Republic" OR Eritrea OR Niger OR Ethiopia OR Rwanda OR "gambia" OR "Sierra Leone" OR "Guinea-Bissau" OR Somalia OR Angola OR Honduras "Papua New Guinea" OR Bangladesh OR India OR Philippines OR Benin OR Jordan OR Samoa OR Bhutan OR Kenya OR "Sao Tome and Principe" OR Bolivia OR Kiribati OR Senegal OR "Cabo Verde" OR "Kyrgyz Republic" OR "Solomon Islands" OR Cambodia OR "Lao PDR" OR "Sri Lanka" OR Cameroon OR Lebanon OR Tajikistan OR Comoros OR Lesotho OR Tanzania OR "Republic of the Congo" OR Mauritania OR "Timor-Leste" OR "Côte d'Ivoire" OR "Federated States of Micronesia" OR Tunisia OR Djibouti OR Morocco OR Uzbekistan OR "Egypt" OR Myanmar OR Vanuatu OR Eswatini OR Nepal OR Vietnam OR Ghana OR Nicaragua OR "West Bank and Gaza" OR Guinea OR Nigeria OR Zambia OR Haiti OR Pakistan OR Zimbabwe) |
| **CINAHL** | ("severe acute respiratory infection*") AND (death* OR “fatal outcome*” OR mortalit*) AND ("resource-limited setting*" OR “resource limited setting*” OR "underdeveloped countr*"OR “under developed countr*” OR "lower-middle income econom*" OR "middle-income countr*" OR "lower middle income econom*" OR "middle income countr*" OR "low-income countr*" OR "low income countr*" OR LMIC OR "low- and middle-income countr*" OR "low and middle income countr*" OR “developing countr*” OR Afghanistan OR "North korea" OR "South Sudan" OR "Burkina Faso" OR Liberia OR Sudan OR Burundi OR Madagascar OR "Syrian Arab Republic" OR "Central African Republic" OR Malawi OR Togo OR Chad OR Mali OR Uganda OR "Democratic Republic of the Congo" OR Mozambique OR "Yemen Republic" OR Eritrea OR Niger OR Ethiopia OR Rwanda OR "gambia" OR "Sierra Leone" OR "Guinea-Bissau" OR Somalia OR Angola OR Honduras "Papua New Guinea" OR Bangladesh OR India OR Philippines OR Benin OR Jordan OR Samoa OR Bhutan OR Kenya OR "Sao Tome and Principe" OR Bolivia OR Kiribati OR Senegal OR "Cabo Verde" OR "Kyrgyz Republic" OR "Solomon Islands" OR Cambodia OR "Lao PDR" OR "Sri Lanka" OR Cameroon OR Lebanon OR Tajikistan OR Comoros OR Lesotho OR Tanzania OR "Republic of the Congo" OR Mauritania OR "Timor-Leste" OR "Côte d'Ivoire" OR "Federated States of Micronesia" OR Tunisia OR Djibouti OR Morocco OR Uzbekistan OR "Egypt" OR Myanmar OR Vanuatu OR Eswatini OR Nepal OR Vietnam OR Ghana OR Nicaragua OR "West Bank and Gaza" OR Guinea OR Nigeria OR Zambia OR Haiti OR Pakistan OR Zimbabwe) |
